# The role of working memory for learning with context-personalized tasks in elementary school

**DOI:** 10.3389/fpsyg.2026.1671810

**Published:** 2026-02-13

**Authors:** Ann-Kathrin Laufs, André Meyer, Maleika Krüger, Sebastian Kempert

**Affiliations:** Primary Education and Research on Learning and Instruction, Department of Primary Education, University of Potsdam, Potsdam, Germany

**Keywords:** instructional design, context personalization, interest, working memory, cognitive load, early science education, control-of-variables strategy

## Abstract

Context personalization is an instructional approach aimed at enhancing students’ engagement and cognitive processing by embedding learning content in familiar contexts. Numerous studies explore the benefits of personalized tasks for learning, but few empirically examine cognitive mechanisms underlying the effects of context personalization. In a cluster-randomized control trial with *N* = 156 elementary school students, we investigated (1) whether context personalization leads to an increased interest in the learning content. Furthermore, we examined (2) the role of working memory for learning and (3) whether the assumed effect of working memory on students’ learning performance was moderated by the use of context-personalized tasks. The results indicate that context personalization elicits interest in the learning content. In addition, working memory was a significant predictor of student performance across conditions. However, the hypothesized moderating effect of context personalization on the relationship between working memory and student performance was not supported. These results contribute to a more nuanced understanding of the cognitive and motivational effects of context-personalized tasks in elementary science education.

## 1 Introduction

Children enter elementary school with large differences in various learning prerequisites, including cognitive, motivational, and language characteristics (Markic and Abels, 2014; Pan et al., 2019). Among these, cognitive factors—particularly working memory and prior knowledge—take on a central role in school-related learning (Alloway, 2006; Alloway and Alloway, 2010; Alloway and Passolunghi, 2011; Gathercole and Alloway, 2004; Simonsmeier et al., 2021). Numerous studies have found evidence that working memory and its different subsystems — i.e., phonological loop, visuospatial sketchpad, central executive — are significant predictors of learning outcomes, for example in the domain of mathematics (overviews in Allen et al., 2019; Friso-van den Bos et al., 2013). Moreover, empirical findings consistently indicate that limitations in the capacity of working memory to store and process information can constrain learning (Baddeley, 1986; Cowan, 2010; Miller, 1956). Yet because working memory capacity is limited, instructional design research has long focused on principles that regulate cognitive load (Chandler and Sweller, 1991; Sweller et al., 1998, 2019). One proposed approach allowing for cognitive facilitation effects involves embedding new learning content in familiar task contexts, which may range from everyday phenomena to topics aligned with students’ personal interests (Walkington and Hayata, 2017). Such context personalization may facilitate learning by linking new knowledge to existing schemas (Goldstone and Son, 2005; Koedinger et al., 2008), but it can also impose additional cognitive demands if “seductive details” distract from the content (Harp and Mayer, 1998).

While context personalization has been examined mainly in mathematics and with secondary school students (Bernacki and Walkington, 2014; Høgheim and Reber, 2015; Walkington, 2013; Fancsali and Ritter, 2014), little is known about its cognitive mechanisms, particularly the role of working memory (for an exception, see van de Weijer-Bergsma and van der Ven, 2021). This lack of evidence is especially notable for elementary science learning, where abstract concepts can be challenging and student interest tends to decline with age (National Research Council, 2012; Tröbst et al., 2016). These factors underscore the importance of investigating how context personalization may support science learning at elementary school level.

We addressed these research gaps by examining how context personalization influences elementary school children’s interest in learning content related to formal scientific thinking. Further, we investigated the role of working memory by analyzing its influence on students’ performance, and, finally, we examined whether the effect of working memory is moderated by the use of context-personalized tasks. As learning content, we chose the control-of-variables strategy (CVS) as it includes experimental design and variable manipulation, key topics in elementary science curricula (Brandenburger et al., 2022; Chen and Klahr, 1999; Edelsbrunner et al., 2018; Ross, 1988; Schwichow et al., 2016).

## 2 Working memory in academic learning

In the field of psychology, several theoretical models explain the structure and function of working memory (Adams et al., 2018; Berti, 2010). The most established in educational psychology is Baddeley’s multi-component model (Baddeley, 1986, 2000, 2012; Baddeley et al., 2019). It distinguishes two subsystems—the phonological loop and the visuospatial sketchpad—controlled by the central executive. The subsystems temporarily store information, hold multiple items simultaneously, and relate them to one another (Baddeley, 2000). The processed information is system-specific: linguistic and acoustic information is processed and stored in the phonological loop, whereas static and dynamic visual information is processed and stored in the visuospatial sketchpad. The central executive is superordinate to these two memory components and performs cross-domain, modality-unspecific control functions, such as allocating cognitive resources, retrieving long-term knowledge, controlling inhibition, and suppressing distracting stimuli irrelevant to the task (Baddeley, 1986; Friedman and Miyake, 2017). Empirical studies show that the subsystems are limited in storage capacity and storage duration and differ individually (Baddeley, 1986; Cowan, 2001, 2010; Miller, 1956).

Working memory is a robust predictor of academic achievement across domains (Alloway and Alloway, 2010; Cortés Pascual et al., 2019; Friso-van den Bos et al., 2013; Gathercole et al., 2004; Gathercole and Alloway, 2004; Hasselhorn, 2005; Klesczewski et al., 2018; Peng and Fuchs, 2016). A cross-national and cross-domain meta-analysis by Cortés Pascual et al. (2019) analyzed the link between working memory and academic performance in elementary school children and found a medium effect size. Gathercole et al. (2004) analyzed the association between working memory and performance on national curriculum assessments in English, mathematics, and science in children aged 7 and 14 years. In the younger group, there was a strong association between working memory and performance in all three domains. In the older group, the association was strong for mathematics and science but not for English (Gathercole et al., 2004). Friso-van den Bos et al. (2013) conducted a meta-analysis on the association between central executive functions and mathematics performance, examining moderators including mathematics test format, working memory test format, and participant age. The results showed positive correlations for all central executive functions, with phonological updating exhibiting the strongest, medium-sized effect, and confirmed the hypothesized interactions of all three moderators. Working memory is also crucial for science learning across developmental stages. Evidence links working memory to science performance in preschool (van der Graaf et al., 2016), elementary (St Clair-Thompson and Gathercole, 2006; Träff et al., 2019; Schäfer et al., 2024), and secondary school students (Donati et al., 2018). Van der Graaf et al. (2016) showed that variance in evidence evaluation—a scientific thinking skill—was explained by verbal working memory, inhibition control, vocabulary, and grammar but not by visuospatial working memory. Schäfer et al. (2024) found that working memory and cognitive flexibility significantly contributed to science problem-solving performance, whereas inhibition did not show an independent effect. Further studies have investigated the links between working memory and different science domains (for physics, see Rhodes et al., 2016, Träff et al., 2019; for biology, see Rhodes et al., 2014). In a longitudinal study, Träff et al. (2019) showed that verbal working memory, logical reasoning, and spatial processing represent significant predictors of later physics achievement.

Although the relationship between individual working memory capacity and learning outcomes varies depending on the measures employed (e.g., fact-learning vs. problem-solving; text-based tasks vs. table-based or visual tasks), the overall positive link between working memory and academic learning appears relatively well established in empirical research. These findings lead to two possible approaches to fostering school-related learning. The first approach would be to train and thus expand children’s working memory capacity. However, training studies aiming at the enhancement of working memory resources have shown no or only very limited effects (Melby-Lervåg et al., 2016; Redick, 2019). The second approach would be to explicitly consider working memory demands in the design of learning materials (Blayney et al., 2015; Sweller, 2020; van Merriënboer et al., 2003). We focus on the latter approach, which raises the central question of how learning materials should be designed, considering the important role of working memory and its interindividual differences. The next chapter introduces cognitive load theory (CLT) as our theoretical framework for understanding cognitive demands in learning processes (Sweller, 1994; Sweller et al., 1998; Sweller et al., 2019). This is followed by an outline of context-personalized learning materials as a strategy to reduce cognitive demands in the learning process and their effects on learning outcomes.

## 3 Consideration of cognitive resources in instructional design

### 3.1 Cognitive load theory

CLT outlines three types of cognitive load: intrinsic, germane, and extraneous cognitive load. Intrinsic cognitive load is characterized by the internal complexity of the learning content; germane cognitive load refers to the mental effort devoted to processing, constructing, and automating schemas during learning, and extraneous cognitive load specifies the requirements that arise from the design of the learning materials (Chandler and Sweller, 1991). Later work has questioned this three-component distinction. Kalyuga (2011), for instance, argued that germane processes are inseparable from intrinsic load and should therefore be subsumed under it. Extending this debate, more recent contributions suggest replacing the notion of germane load with that of germane resources, emphasizing that what is at stake is not an additional type of load but rather the allocation of available working memory capacity to learning-relevant activities (Greenberg and Zheng, 2022).

Designing learning materials allows for manipulation of extraneous cognitive load: Learning materials that include irrelevant information such as additional illustrations or text can create high extraneous cognitive load and are therefore thought to hinder learning (Harp and Mayer, 1998). In contrast, low extraneous cognitive load is assumed to be favorable for learning as it allows learners to allocate their germane resources more efficiently to the actual learning process (Chandler and Sweller, 1991). Several design principles have emerged from the empirical research on cognitive load (Sweller, 2020; van Merriënboer and Sweller, 2005). Findings indicate that learners with low prior knowledge may benefit from well-designed instructional materials that provide additional cognitive support, such as guided instruction. However, learners with high prior knowledge may perceive this additional information to be redundant and therefore experience increased cognitive load, known as the expertise reversal effect (Kalyuga et al., 1998; Kalyuga et al., 2003; Kalyuga, 2007). These findings highlight the importance of considering individual learners’ prior knowledge in instructional design. In our study, we aim to reduce extraneous cognitive load through well-designed context personalization and to foster schema integration by linking new content to learners’ prior knowledge. In this way, the intervention supports the effective use of germane resources, enabling learners—depending on their working memory capacity—to allocate cognitive resources more productively to learning-relevant processes and to process the material more efficiently.

### 3.2 The role of interest in context personalization

One instructional approach that builds on the individual’s prior knowledge and experience is context personalization (Walkington, 2017). Here, learning content is embedded in student-related experiences, everyday phenomena, or individual interests (Walkington, 2013). Individual interests are assumed to be highly effective task contexts due to their cognitive, emotional, and value-related characteristics (Renninger and Hidi, 2016). Empirical evidence indicates that higher levels of interest are positively associated with learning outcomes and achievement (Schiefele et al., 1993; Jansen et al., 2016). Furthermore, interest affects learning through multiple psychological mechanisms, including increased attention, deeper cognitive elaboration, and sustained effort during task engagement (Ainley et al., 2002; Harackiewicz et al., 2016; Krapp, 1999). Following the four-phase model of interest development (Hidi and Renninger, 2006), each phase of interest development is associated with a varying amount of prior knowledge, positive emotions, and subjective value (Hidi and Renninger, 2006; Krapp and Prenzel, 2011; Renninger and Hidi, 2016; Schiefele, 1991). In the first phase, the triggered situational interest, learners experience a short-lived interest prompted by external factors, such as surprising instructional information or a group work setting (Hidi and Renninger, 2006; Renninger and Hidi, 2016). As learners continue to engage with the content, their interest transitions to the second phase, the maintained situational interest, characterized by personal engagement and attention (Hidi and Renninger, 2006; Prenzel et al., 1986). The third phase, emerging individual interest, is characterized by reengagement with content, accompanied by positive emotions, prior knowledge, and personal value. In the fourth phase, well-developed individual interest, the degree of prior knowledge, positive emotions, and subjective value increases, as individuals connect their interest to their identity, values, and goals (Hidi and Renninger, 2006). When educational material aligns with the learner’s pre-existing individual interest, the emerging state is defined as actualized individual interest (Renninger and Hidi, 2016; Schraw and Lehman, 2001). Various studies have shown that context personalization has the potential to trigger situational interest in the task at hand (Bernacki and Walkington, 2018; Bernacki and Walkington, 2014; Høgheim and Reber, 2015). However, the mere elicitation of interest—while important—is not sufficient to facilitate learning. In this regard, the cognitive benefits of context personalization become relevant: personalized contexts may reduce extraneous cognitive load and foster deeper processing, particularly when they draw on learners’ existing knowledge (Paas et al., 2010; Sweller et al., 2019).

To illustrate different implementations of context personalization, and how they may differentially activate prior knowledge, evoke positive emotions, and enhance subjective value, we present two instructional examples related to the teaching of measurement units. In the first, the length of the students’ route to school is used as a task context connected to their personal experience. Although learners may perceive personal relevance and increased attention toward the task at hand, they are not expected to have a particular high level of individual interest or prior knowledge. In contrast, a second scenario involves a topic of well-developed individual interest— for instance, soccer—used to contextualize measurement tasks such as penalty or corner kick distances (Skutella and Eilerts, 2018). This context is thought to enable learners to connect new information to existing mental models, scripts, or schemas (Goldstone and Son, 2005) and therefore facilitate a deeper understanding of measurement units. Embedding new learning content in such a familiar and personally meaningful context may reduce the number of unknown surface features—such as unfamiliar terms, settings, or thematic elements—that learners must process in addition to the instructional content itself. According to cognitive load theory, this reduction in extraneous cognitive load (Paas et al., 2010; Sweller et al., 2019) can free up cognitive resources for the processing of core conceptual information. To understand how different ways of implementing context personalization affect students’ task interest and learning outcomes, in the following, we discuss dimensions of instructional design (Walkington and Hayata, 2017) and present empirical findings on context personalization.

### 3.3 Effects of context personalization on students’ performance

Walkington and Hayata (2017) conceptualize context personalization along three interacting instructional design dimensions: (1) The design dimension depth describes the degree of authentic connection between the learning content and students’ interests. (2) Grain size describes the specificity of the interest-based connections. (3) Ownership defines the student’s role in context personalization (Bernacki and Walkington, 2018; Walkington and Bernacki, 2020; Walkington and Hayata, 2017). A possible fourth design dimension, which has not yet been fully integrated into the framework, is the richness of the learning material. Richness describes the amount of context information that is not necessary for the learning process (Walkington and Hayata, 2017). These dimensions serve to systematize research on context personalization and clarify why its effects on performance have yielded inconsistent results.

Cognitive-motivational (facilitation) effects arise depending on how these design dimensions are used in the implementation of context personalization (Walkington and Hayata, 2017). In addition to implementation, the different measures of learning outcomes, in particular, students’ performance, and the individual students’ cognitive resources play a role in classifying the findings. Learning material that considers the design dimensions for context personalization is associated with stronger positive effects on performance (for implementation examples, see Bernacki and Walkington, 2014). Following Goldstone and Son (2005), context personalization can facilitate the connection of prior knowledge with new information in long-term memory and, hence, avoid misconceptions (Goldstone and Son, 2005).

In the domain of mathematics, empirical evidence shows that a medium level of context personalization as defined in Walkington and Hayata’s (2017) design dimensions supports learning better than superficial and coarse-grained context personalization. Bernacki and Walkington (2014) found that students’ individual interest in mathematics moderates the effects of deep personalization, showing higher learning gains for students with low interest in mathematics. In an investigation of context personalization as instructional assistance in secondary school algebra classes, Walkington and Maull (2011) reported an interaction effect between the student level and the problem level in line with the expertise reversal effect: They found context personalization to enhance the performance of both low-performing and high-performing students on complex problem-solving tasks. However, high-performing students demonstrated a decline in performance on personalized, relatively simple problem-solving tasks. A further study supports these findings, showing that low-performing eighth-grade students benefiting the most from personalized mathematics classes in terms of posing, solving, and sharing algebra problems related to their interests (Walkington, 2017).

Further, Walkington et al. (2016) reported that context personalization—achieved by personalizing tasks based on a student interest interview and combined with student choice (medium context personalization)—positively affected learning outcomes. They also found that replacing single words with terms related to an area of student interest (superficial context personalization) had no effect on mathematical learning (Walkington et al., 2016). There is evidence that superficial and coarse-grained context personalization, such as personalizing only the names of the characters in the instruction for a task, does not lead to contextual grounding and is likely to result in a null effect on performance (Høgheim and Reber, 2015; Bates and Wiest, 2004). However, very rich context personalization can also impede learning, as it may include seductive details that distract from the actual learning objective (seduction hypothesis) or negatively influence information processing (disruption hypothesis) (Harp and Mayer, 1998; Mayer et al., 2008; Rey, 2012).

## 4 Method

### 4.1 Research questions and hypotheses

The theoretical considerations and existing empirical findings show that context personalization does not always lead to the desired cognitive effects on learning. Explanations of the contradictory evidence point to instructional design features, individual learning prerequisites and their relationships. To better understand how context personalization approaches can be used in elementary school, we conducted an empirical intervention study with elementary school students. Our study thus expands the scope of research in the field of context personalization beyond the predominant focus on mathematical problem-solving tasks in secondary school. Building on the theoretical assumptions and empirical findings outlined above, we investigate the following research questions and hypotheses: (1) Does the use of context-personalized tasks increase students’ interest in the learning content? (2) To what extent do different components of working memory—namely phonological, visuospatial, and central executive—predict students’ performance in terms of control-of-variables strategy comprehension? (3) Does the use of context-personalized tasks have a (moderating) effect on the relationship between working memory and student’s performance, indicating an aptitude–treatment interaction?

Considering the design principles of Walkington and Hayata (2017) in the construction of learning tasks, we classify our intervention as medium-level context personalization, which is expected to facilitate learning effects. Accordingly, we formulated our hypotheses in a positive direction. Based on previous findings indicating that context personalization increases situational interest in the learning content (Bernacki and Walkington, 2018; Høgheim and Reber, 2015), we assumed that a context-personalized condition elicits higher interest in the task context than a standard condition. Moreover, we expected that interest in the task context carries over to interest in the learning content. We therefore expected to find a group difference between a personalized condition and a control condition in terms of interest in the learning content that would be explained by interest in the task context. Drawing on established theoretical frameworks and empirical evidence concerning the role of working memory in academic learning (Alloway and Alloway, 2010; Rhodes et al., 2016), we assumed that the three different working memory components are associated with students’ performance. However, due to the high proportion of language in the instructions and the text-based structure of the tasks, we expected the phonological working memory component to be most predictive. Further, we assumed that functions of the central executive, such as suppressing distracting stimuli irrelevant to the task, also predict performance. We expected only a weak association between the visuospatial working memory component and the dependent variable as the central information and the task problem could be taken exclusively from the verbal instructions or the text. We hypothesized that the predictive role of working memory for performance is moderated by the use of context-personalized tasks, resulting in an aptitude-treatment interaction (Koran and Koran, 1984). This interaction suggests that the effectiveness of instructional methods depends on the learner’s specific characteristics—which in our study are interest and working memory resources. Based on findings on the expertise reversal effect (Kalyuga, 2007) and the positive effects of contextual grounding (Guida et al., 2009), we assumed that low working memory capacity may partly be compensated by the use of context-personalized tasks. Learning a new concept in a familiar and interesting context should hence reduce working memory demands, as the processing of new information is facilitated by the use of familiar surface features. We hypothesized that students with high or intermediate levels of working memory, in contrast, do not benefit substantially from personalized tasks.

### 4.2 Study design and intervention procedure

We conducted cluster-randomized trial of a personalized learning intervention with a pre- and post-test at six elementary schools located in urban and suburban regions of Germany. The Berlin Senate Department for Education, Youth and Family and the Ministry of Education Youth and Sports of the State of Brandenburg evaluated our study design and approved all instruments used in this study. We obtained prior informed consent from parents. Participation in the study was voluntary and participants could withdraw at any time. We processed the collected data using pseudonyms at all times.

**Figure 1:**
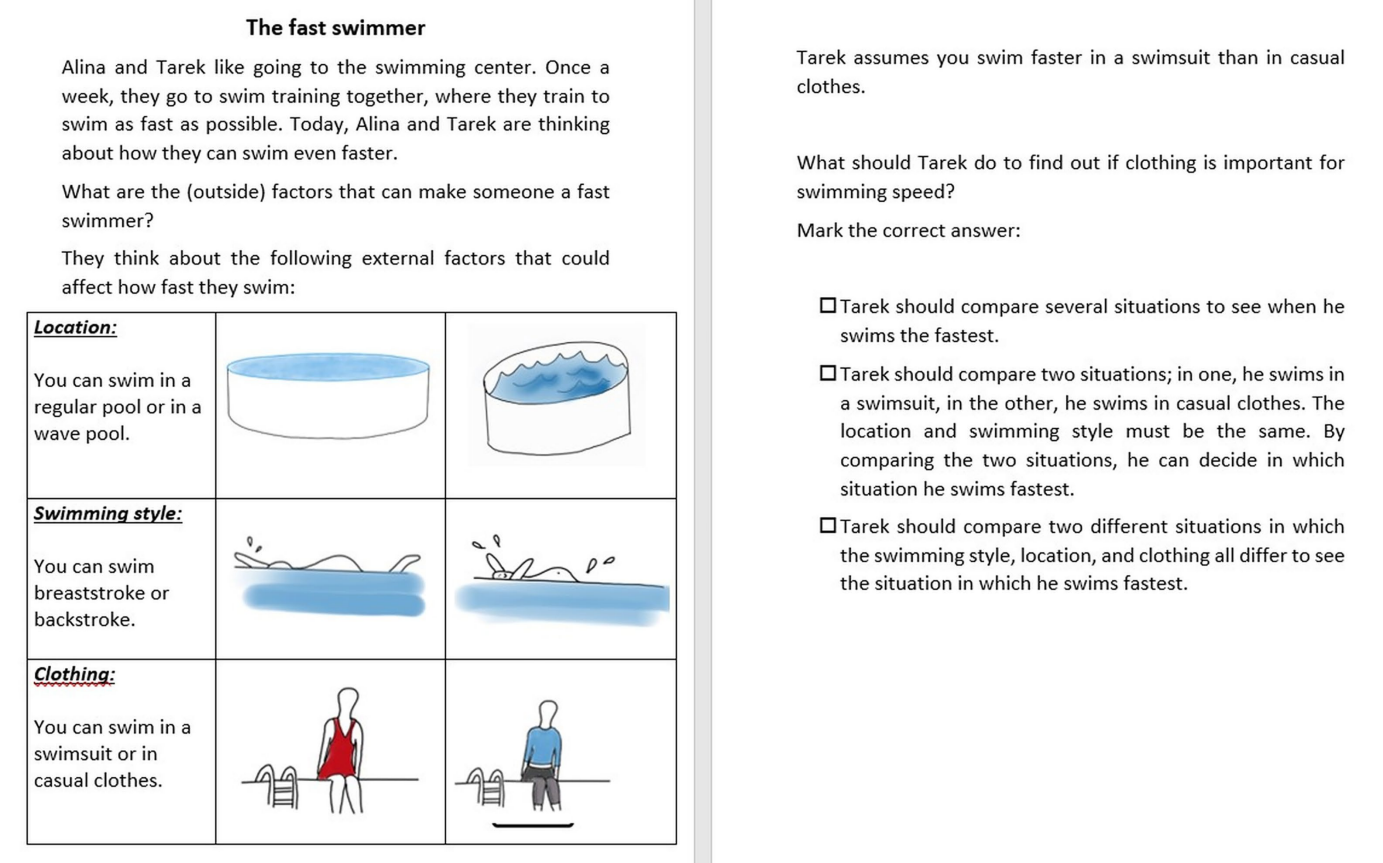
Context-personalized worksheet for the CVS intervention session (figures prepared by a student assistant).

The main intervention goal was to teach elementary school children the control-of-variables strategy as a basic experimentation strategy enabling them to identify and describe causal relationships in an experiment with multiple variables (Chen and Klahr, 1999). The core function of the CVS lies in manipulating a variable of interest while keeping all other variables constant, resulting in a unconfounded experimental setting. The application and understanding of the CVS are important to formal scientific thinking and therefore relevant to the elementary school curriculum (Sodian and Mayer, 2013). CVS can be taught through hands-on and paper-based activities (Schwichow et al., 2016; Ross, 1988). In the present study, CVS was taught and tested through paper-based activities. Figure 1 gives an example of a worksheet for teaching the CVS in the context of swimming.

The study consisted of six sessions, each lasting 50 min, conducted over an eight-week period. Trained test administrators, who were teacher trainees or psychology students, facilitated the sessions during regular school hours. During the first meeting held in the class, we introduced the project, and all the students completed a paper-based questionnaire to assess their prior knowledge of the CVS (Edelsbrunner et al., 2018) and their basic cognitive abilities (Weiß, 2006). After an average of 4 days, we met with each student again individually. In this second meeting, we gathered the child’s socio-demographic information through conversation. Next, the student completed a computer-based assessment of their working memory capacity (Hasselhorn et al., 2012). Following this, we administered an adaptive, researcher-developed computer-based questionnaire on students’ interests, based on relevant interest studies in the German-speaking context (e.g., Pruisken, 2005; Wolfert and Pupeter, 2018) and our own preliminary study (Laufs and Kempert, 2023). Students could choose one or more of six superordinate topic areas (sports, music, indoor activities, animals, technology, playing games). Subsequently, we presented them with an average of five specific items of interest based on the areas they selected. For example, students who selected sports saw options like playing soccer, playing basketball, playing handball, riding horses, bicycling, swimming, and doing gymnastics. Based on their interest ratings, we then assigned students to small groups of four on average. The criterion for inclusion in an interest group was a strong interest across all interest components (M ≥ 3) as measured by the interest questionnaire on a 4-point Likert scale. Each group was randomly assigned to one of the conditions during the intervention: Groups in the personalized condition received tasks with a context based on their individual interests (e.g., soccer). In the control condition, magnetism served as the task context which may be regarded as a standard context in German early science classes (see Berlin Senate Department for Education, Youth and Family and Ministry of Education, Youth and Sports of the State of Brandenburg, 2015). Due to the abstract nature of the concept of magnetism, we regarded it as not particularly interesting for elementary school children. Although we tried to assign students to the conditions randomly, rigorous randomization was not possible for practical and organizational reasons (e.g., the existence of remedial school classes). The result was a total of 28 groups in the personalized condition (task contexts: playing soccer, reading, swimming, riding horses, doing gymnastics, bicycling) and 13 groups in the control condition with magnetism as a task context. The intervention was set for the third and fourth meetings, which were held 4 days apart on average.

The tasks assigned to the participants in the personalized and control conditions differed only with respect to their thematic embedding; the underlying task and sentence structure were identical: All sessions consisted of the following phases: (1) general introduction, (2) introduction to the task with a worksheet, (3) explanation of comparisons (in general), (4) explanation of “fair” comparisons, (5) application and practice of “fair” comparisons together on Example 1, (6) application and practice together on Example 2, (7) review of results.

Phase 1 started with a segment welcoming the students. In addition, for the personalized condition, there was a short conversation that addressed students’ interests from the interest questionnaire. Phase 2 introduced the thematic starting point for dealing with “fair” comparisons in the form of a paper-based problem task. In the personalized condition, the material referred to the students’ individual interests. In the case of swimming, students received the following problem: “What are the (external) factors that can make someone a fast swimmer?.” In a guided group discussion, participants and the test administrator talked about what factors might play a role (e.g., location—a pool with or without waves; swimming style—breaststroke or backstroke; clothing—swimsuit or casual clothes). In the control condition, the material referred to the topic of magnetism: “Why is it that some magnets are stronger (meaning they can pick up more nails) than other magnets?.” In Phases 3 and 4, the test administrator and students jointly developed an understanding of comparisons in general and “fair” comparisons. Different comparisons were drawn to help the students double-check their presumptions as part of the task (e.g., “I guess it is because of the clothes!”). As an extension, Phase 4 introduced the fair comparison, which focuses on only one possible factor (e.g., comparison of swimsuits vs. casual clothes) and holds all other factors constant (e.g., location and swimming style). In Phases 5, 6, and 7, students practiced applying the CVS to examples and discussed and reviewed their results.

Responses on the worksheets differed between the two intervention sessions. For Session A, students were presented with a multiple-choice format with three response options. Session B presented students with an open response format, including two pictures (confounded vs. non-confounded comparison). Students were required to provide a written argumentation for their choice. At the end of both meetings, we surveyed the situational interest in the task context and the learning content CVS and measured reading comprehension (ELFE II, Lenhard et al., 2018). After each intervention session, the test administrators completed a brief qualitative observation form to evaluate the implementation of the intervention according to the intervention instructions, aiming to check treatment fidelity (Sanetti et al., 2021). We assessed CVS comprehension in a post-test 2 weeks later (Edelsbrunner et al., 2018). To ensure that the CVS comprehension measurement focused on the conceptual understanding of the material rather than reading ability, test administrators read the instructions and the individual items of the pre-test and post-test on CVS comprehension and the questionnaire aloud to record the interest in the task context and the learning content CVS.

### 4.3 Sample

The sample consists of *N* = 185 elementary school students from urban and suburban areas in Germany. We excluded 29 students with missing data on all sum scores for pre-and post-tests and the grouping variable. These students were registered for the study but could not participate due to remedial classes or illness. In the process of participant recruitment, we asked the classroom teachers whether they had already introduced the learning content CVS. This would have been an exclusion criterion for the respective class. This resulted in a final sample of *n* = 156 students for the present analysis (personalized condition: 100, control condition: 56). The students were *M* = 8.9 (*SD* = 0.77) years old, and 48.7% of students reported being female.

### 4.4 Instruments

Table 1 provides an overview of the instruments and test measures used to assess students’ CVS comprehension, working memory, situational interest, cognitive abilities, and reading comprehension. A detailed description of each measure can be found below. In the “test session” column, the specific session during which the test or material was administered can be identified out of the total of six sessions.

**Table 1:** Overview of instruments, test measures, ranges, and test sessions.

Concept	Instrument	Subtests	Indices	Overall	Range	Test session^a^
CVS comprehension	CVS Test (Edelsbrunner et al., 2018)	-	-	Overall measure	0–11	1 Pre-test
						5 Post-test
						Working memory capacity	AGTB–5–12 (Hasselhorn et al., 2012)			Overall measure including six subtests	−2.1–2.1 (z-standardized)	2
	Monosyllabic word span test	Phonological component				
	Fantasy word test	Phonological component				
	Matrix	Visuospatial component				
	Corsi block	Visuospatial component				
	Object span	Central executive				
	Digits backward	Central executive				
Situational interest	Researcher-developed (based on Ainley et al., 2002)			-	1–4	3 4		Interest in the task context	Task context				Interest in the CVS	Learning content CVS
	Cognitive abilities	CFT–20–R (Weiß, 2006)	Short version	**-**	Overall measure	t-standardized	1
Reading comprehension	ELFE II (Lenhard et al., 2018)	Text comprehension	**-**	Overall measure	0–26	3

a Session in which the test was administered (1–5).

#### 4.4.1 CVS comprehension

For the assessment of CVS comprehension at the two points of measurement (pre-test and post-test), we employed an instrument developed by Edelsbrunner et al. (2018) that consists of a set of established tasks specifically designed to evaluate understanding of the CVS. To streamline the test duration and prevent thematic overlap with the intervention session’s areas of interest, we selected nine out of the total of 14 test items. Of these, seven were in multiple-choice format with three response options and the remaining two were in an open response format. Each correct answer in the multiple-choice tasks was scored as one point, whereas open-ended response tasks were assigned a maximum of two points. Thus, the maximum score was 11 points. To ensure reliability and minimize bias, two independent raters assessed the open-ended responses, achieving a satisfactory interrater reliability (Cohen’s *κ* = 0.81) (Wirtz and Kutschmann, 2002). The internal consistency of the shortened test version, measured by Cronbach’s *α*, was moderate at 0.64, which can be partially attributed to the different task formats employed.

#### 4.4.2 Working memory

To assess working memory capacity, we used the Working Memory Test Battery for children aged 5 to 12 years (in German Arbeitsgedächtnistestbatterie AGTB 5–12, Hasselhorn et al., 2012). The AGTB 5–12 is a standardized and computer-based test composed of a total of 12 subtests that separately assess the theoretically hypothesized components of Baddeley’s (1986) multi-component model of working memory. According to Hasselhorn et al. (2012), the 12 subtests are designed for conducting individual diagnostics, whereas the screening version is comprised of six subtests. The screening version is valuable for research purposes due to its time-efficient and valid coverage of the phonological and the visuospatial component as well as the central executive. Following Hasselhorn et al. (2012), we used the screening version of the AGTB 5–12, substituting only one subtest to assess the overall capacity of the phonological loop. To measure the phonological component, we applied the monosyllabic word span test (α = 0.95) and the non-word-repetition test (*α* = 0.74). Whereas the word span test assesses the overall capacity of the phonological loop, the non-word-repetition test surveys the extent of phonetic memory and processing precision (Hasselhorn et al., 2012). We assessed the capacity of the visuospatial component using the subtests matrix (visual-static component) (*α* = 0.99) and corsi block (spatial-dynamic component) (*α* = 0.96). We assessed the central executive component using two subtests: The first of these, the object span test (*α* = 0.96), requires the storage and simultaneous processing of information and thus measures the coordination capacity of the central executive. The second of these, the digits backwards test (*α* = 0.96), focuses on coordination capacity in controlling encoding and retrieval strategies of temporarily stored information (Hasselhorn et al., 2012). The computer-based nature of the task instructions and the evaluation procedures ensure a high level of objectivity. Since the individual subtests use different units of measurement, that is, the number of words recalled or processing time in seconds, we z-standardized all values.

#### 4.4.3 Interest in the task context and learning content (CVS)

We developed a paper-based questionnaire to measure actualized individual interest in the task context and situational interest in the learning content CVS. Each scale consists of six items assessed on a four-point Likert scale visualized using emoticons (1 = 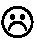, i.e., a sad face; 4 = 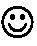, i.e., a happy face) supported by verbalization (1 = *I strongly disagree*; 4 = *I strongly agree*) (adapted from: Ainley et al., 2002). Table 2 outlines our assessment of the cognitive component, the affective component, and the value-related component for the specific task context and also for the CVS. The internal consistency of the scale context was considered good (Cronbach’s α = 0.87), whereas the scale learning content showed satisfactory internal consistency (Cronbach’s α = 0.79). The retest reliabilities for the scales context (*r* = 0.82) and learning content (*r* = 0.70) were rated as good and satisfactory, respectively.

**Table 2:** Overview of items to assess interest in the task context and the learning content (CVS).

Interest component	Item	Adapted from
Affective component	I enjoy engaging with the topic of XY. When I deal with the topic XY, I forget everything around me.	Csiksentmihalyi and Schiefele (1993)
Value-related component	The topic of XY is important to me personally. I would like to learn more about the topic XY.	Ainley et al. (2002)
Cognitive component	I already know a lot about the topic XY. I know things about the topic XY that others do not know.	Ainley et al. (2002)

#### 4.4.4 Control variables

We conducted a standardized test of reading comprehension for Grades 1 to 7 using the subtest text comprehension of the ELFE II test to control for language-related differences (Lenhard et al., 2018). The subtest consists of 26 short texts followed by multiple-choice questions. Each correct answer is scored with one point; thus, the maximum score was 26 points. The subtest shows high retest reliability (*r* = 0.93). Furthermore, we used the short version of the Cattel’s Fluid Intelligence Test, scale 2 (CFT20–R; Weiß, 2006) to control for the influence of basic cognitive skills. This language-free standardized test with satisfactory retest reliability (*rtt* = 0.80) uses a series of graphic items with four different task types: series continuation, classification, matrices, and topological conclusions. To specify the results, we used the t-values generated using the norm sample. We collected demographic information such as age, gender, and linguistic background using a brief questionnaire. Additionally, after each session, the test administrators completed a brief observation form detailing how the intervention was carried out and noting any disruptions or peculiarities. This gave us insight into the implementation and served as a qualitative assessment of treatment fidelity (Sanetti et al., 2021).

#### 4.5 Missing data

After excluding students with missing data on all sum scores and the grouping variable, the final sample consisted of *n* = 156 students (personalized condition: 100, control condition: 56). Table 3 shows the percentages of missing data for each variable, which ranged from 3.8 to 32.1%. Information for the grouping variable was complete in all cases. The missing data are assumed to be missing at random (MAR), as their occurrence is plausibly attributable to external, non-systematic factors such as students’ illness or attendance at remedial classes. To investigate the missing data mechanism, we applied Little’s MCAR test (Little, 1988) using IBM SPSS Statistics software. The MCAR test yielded a non-significant result (χ^2^ = 194.78, *df* = 186, *p* > 0.05), indicating that no systematic differences in the available data patterns could be statistically detected. However, as emphasized in the methodological literature (see Rhoads, 2012), a non-significant result does not prove that data are missing completely at random, it provides no empirical evidence against this assumption. Given the absence of detectable systematic variation in missingness and the lack of any theoretical rationale for assuming systematic missingness, the test results support the use of estimation methods that are robust under MCAR. Missing data were handled using multilevel multiple imputation with the mice package in R (van Buuren and Groothuis-Oudshoorn, 2011). We generated 100 imputed datasets with 50 iterations each. The clustering structure was accounted for by specifying it as the level-2 grouping variable in the predictor matrix. Imputation models were specified manually to reflect hypothesized relationships between variables.

**Table 3:** Missing rates for variables condition in percentage.

Variable	Missing rate^a^		
	Personalized condition (*N* = 100)	Control condition (*N* = 56)	Total (*N* = 156)
Phonological component	11.00	17.90	13.50
Visuospatial component	11.00	17.90	13.50
Central executive	11.00	17.90	13.50
Cognitive abilities	9.00	14.30	10.90
Reading comprehension	2.00	7.10	3.80
Interest task context	25.00	32.10	23.70
Interest CVS	20.00	30.40	27.60
Pre-test CVS	3.00	5.40	3.80
Post-test CVS	8.00	14.30	10.30

a Percent values.

#### 4.6 Analyses

Given the cluster-randomized design of the study, in which students were assigned to small groups based on their interest ratings and subsequently exposed to either a personalized learning intervention or a control condition, we treated the group as the unit of randomization. In total, 41 small groups were formed. Accordingly, the data were analyzed using statistical models that account for the clustered structure, assuming potential intraclass correlations among students within the same group due to shared experiences, peer interactions, and potentially similar environmental influences. Due to the relatively small number of clusters, cluster-robust standard errors were applied to ensure valid inference (Huang and Li, 2022). All statistical analyses were conducted using R.

To address Research Question 1, we employed a hierarchical regression model to assess the incremental contribution of condition and interest in the task context to interest in the learning content. For Research Question 2, we conducted distinct linear regression models to investigate how three working memory components—phonological, visuospatial, and central executive—predict post-test performance. In the initial phase of our analysis regarding research question 3, we assessed the potential for multicollinearity among the predictor variables. This was done by calculating the variance inflation factor (VIF) scores for each variable. The VIF scores obtained were slightly above 1, indicating that there is no significant multicollinearity present in our data (Shrestha, 2020). This suggests that the predictor variables are sufficiently independent of each other, and multicollinearity is not a concern for our model. Subsequently, we employed a regression model that included an interaction term to explore how working memory and interest interact as moderators, potentially influencing post-test performance as the outcome variable. To maintain the clarity of the model, we aggregated the three working memory components into a single global value.

## 5 Results

### 5.1 Descriptive results

Table 4 shows the mean scores and standard deviations for the pre-test on CVS, the z-standardized measure for working memory components, the standardized t-scores for cognitive abilities, and reading comprehension by condition. Regression analysis with condition as a dummy variable did not reveal any significant differences between the two conditions for the given scores (*p* > 0.05).

**Table 4:** Means and standard deviations by condition.

Instrument	Comprehension of CVS pre-test	Phonological component	Visuospatial component	Central executive	Cognitive abilities	Reading comprehension
(min. – max.)	0–11	z-standardized	z-standardized	z-standardized	t-standardized	0–26
Personalized condition (*N* = 100)	4.21 (2.30)	0.55 (0.79)	−0.14 (0.75)	−0.08 (0.84)	55.58 (10.52)	10.43 (4.66)
Control condition (*N* = 56)	3.71 (2.19)	0.377 (0.95)	−0.36 (0.85)	−0.33 (0.88)	53.87 (10.21)	9.52 (4.55)
Total (*N* = 156)	4.03 (2.27)	0.49 (0.85)	−0.22 (0.80)	−0.17 (0.86)	54.97 (10.42)	10.10 (4.62)

An overview of the correlation coefficients for all study variables is provided in Table 5. Although working memory and basic cognitive abilities are distinct constructs, the correlation between these two variables is well established (Alloway and Alloway, 2010; Friedman and Miyake, 2017) and also evident in our data. Therefore, we included basic cognitive abilities in the imputation model but did not use them as a control variable in further analyses.

**Table 5:** Correlation coefficients and standard errors.

Variable	1	2	3	4	5	6	7	8	9	10
1 Cognitive abilities	1									
2 Interest CVS	0.132 (0.105)	1								
3 Interest task context	−0.034 (0.095)	0.677*** (0.070)	1							
4 Phonological component	0.315*** (0.061)	0.021 (0.095)	0.045 (0.093)	1						
5 Visuospatial component	0.435*** (0.069)***	0.232* (0.103)	0.014 (0.116)	0.260*** (0.074)	1					
6 Central executive	0.404*** (0.067)	0.167* (0.085)	−0.002 (0.094)	0.447*** (0.074)	0.539*** (0.063)	1				
7 Reading comprehension	0.532*** (0.062)	0.086 (0.106)	−0.034 (0.109)	0.465*** (0.069)	0.245*** (0.073)	0.408*** (0.063)	1			
8 Pre-test CVS	0.282*** (0.074)	0.174 (0.095)	0.021 (0.092)	0.326*** (0.057)	0.209** (0.073)	0.313*** (0.075)	0.467*** (0.064)	1		
9 Post-test CVS	0.420*** (0.065)	0.123 (0.081)	−0.072 (0.087)	0.439*** (0.077)	0.271*** (0.078)	0.367*** (0.082)	0.519*** (0.069)	0.484*** (0.062)	1	
10 Follow-up-test CVS	0.399*** (0.063)	−0.032 (0.087)	−0.213* (0.102)	0.366*** (0.066)	0.207** (0.072)	0.332*** (0.085)	0.468*** (0.078)	0.528*** (0.063)	0.655*** (0.064)	1

***p ≤ 0.001, **p ≤ 0.01, *p ≤ 0.05.

### 5.2 Do context-personalized tasks increase interest in the learning content?

Research Question 1 examined the relationship between condition, interest in the task context, and the interest in the learning content. We hypothesized that the students in the personalized condition would report higher interest in the learning content CVS compared to those in the control condition, and we assumed that this difference could be explained by the interest in the task context. Table 6 shows the means and standard deviations for the interest ratings for the two conditions and overall.

**Table 6:** Means and standard deviations for interest in task context and interest in learning content CVS by condition.

Condition	Interest task context^a^	Interest learning content CVS^a^
Personalized condition (*N* = 100)	3.09 (0.65)	3.12 (0.65)
Control condition (*N* = 56)	2.15 (0.74)	2.52 (0.76)
Total (*N* = 156)	2.75 (0.82)	2.91 (0.75)

a Four-point Likert scale.

As the descriptive results in Table 6 demonstrate, students in the personalized condition reported higher interest in both the task context and the learning content CVS. Results of a regression analysis in Table 7 show a significant effect of the condition on interest in the learning content CVS. In Model 2, we included interest in the task context as a predictor. As the added variable reduces the effect of the condition, the higher interest of the personalized condition relative to the control condition in the learning content CVS can be attributed to interest in the task context.

**Table 7:** Interest in the concept CVS (DV) on condition and interest in the task context.

Predictor	Model 1			Model 2		
	*B*	*CRSE*	*df_BM_*	*B*	*CRSE*	*df_BM_*
(Intercept)	2.439***	0.149	11.55	1.129***	0.182	20.71
Group (0 = CG)	0.713***	0.171	24.44	0.081	0.154	23.36
Interest task context				0. 630***	0.069	23.71
R^2^(adj.)		0.176			0.495	

*** *p* ≤ 0.001, ** *p* ≤ 0.01, * *p* ≤ 0.05, Cluster-robust standard errors (CRSE) calculated with CR2 correction.

### 5.3 Do the different components of working memory (a: phonological, b: visuospatial, c: central executive) predict students’ performance?

To further investigate our second research question, we conducted linear regression analyses with post-test performance on CVS as the dependent variable. In each analysis step, we included the pre-test score of CVS and one of the three working memory indices as predictor. The results in Table 8 showed significant effects for the phonological and the central executive indices on post-test performance while controlling for pre-test performance, which also shows a positive effect on post-test performance. Among the three working memory components, the phonological component is the most predictive of post-test performance, whereas the visuospatial component did not show any significant predictive power.

**Table 8:** Post-test CVS (DV) on pre-test CVS, phonological and visuospatial component, central executive, working memory, condition, and interaction terms.

Predictor	Model 1			Model 2			Model 3			Model 4			Model 5		
	*B*	*CRSE*	*df_BM_*	*B*	*CRSE*	*df_BM_*	*B*	*CRSE*	*df_BM_*	*B*	*CRSE*	*df_BM_*	*B*	*CRSE*	*df_BM_*
(Intercept)	4.445***	0.404	27.42	4.698***	0.453	26.99	4.853***	0.458	26.54	4.831***	0.420	26.76	4.763***	0.580	17.87
Pre-test CVS	0.549***	0.075	21.43	0.593***	0.081	21.96	0.554***	0.081	22.96	0.521***	0.080	22.66	0.518***	0.078	22.98
Phonological WM	0.300*	0.194	18.59												
Visuospatial WM				0.487	0.247	23.49									
Central executive WM							0.654*	0.265	22.37						
Working memory										1.104**	0.306	21.70	1.116*	0.422	7.91
Condition													0.124	0.514	24.46
Working memory X Condition													−0.020	0.575	18.70
R^2^(adj.)		0.279			0.244			0.259			0.290			0.283	

*** *p* ≤ 0.001, ** *p* ≤ 0.01, * *p* ≤ 0.05; Cluster-robust standard errors (CRSE) calculated with CR2 correction.

### 5.4 Does the use of context-personalized tasks have a (moderating) effect on the relationship between working memory and students’ performance?

To examine whether the effect of working memory on post-test performance was moderated by the use of context-personalized tasks, we included the intervention condition and its interactions with working memory as predictors in a model. In order to mitigate issues related to limited test power, all three subscales of working memory were integrated into a global value.

Although the pre-test on CVS and the global value for working memory sustained their predictive power, we found no effect of the intervention condition. Table 8 shows that the interaction effects of working memory and the intervention condition on post-test performance remained non-significant.

## 6 Discussion

In the present study, we investigated the role of context-personalized tasks in the domain of early science education, taking individual working memory resources into account. To this end, we analyzed the effects of an intervention on interest in a selected learning content from the area of formal scientific thinking, namely the CVS (Research Question 1). In addition, we examined the predictive power of the three working memory components (phonological, visuospatial, central executive) on CVS comprehension (Research Question 2). Finally, we tested a possible aptitude-treatment interaction, that is, the interplay of working memory, the use of context-personalized tasks (i.e., condition), and students’ performance (Research Question 3).

Regarding Research Question 1, previous studies showed that context personalization has the potential to increase the situational interest of middle school students in academic learning contents (Bernacki and Walkington, 2018; Høgheim and Reber, 2015; Reber et al., 2018). Our data support this finding for elementary school students: When compared to the theoretical average on the rating scale and the interest ratings of children in the personalized condition for their respective context, the children in the control condition showed lower levels of interest in the context of magnetism.

Consequently, children in the personalized condition outperformed children in the control condition in terms of their interest in the learning content CVS, which is explained by the interest in the task context. In summary, the results support our first hypothesis and align with previous findings (Bernacki and Walkington, 2018; Bernacki and Walkington, 2014; Høgheim and Reber, 2015). Given the positive consequences of interest reported in the literature, such as higher cognitive, affective, and value-related engagement (Hidi and Renninger, 2006; Krapp and Prenzel, 2011; Renninger and Hidi, 2016), this finding has important implications for the design of learning materials and highlights the potential of personalized context to address the decline in students’ interest in science (Tröbst et al., 2016).

The results of Research Question 2 underscore the crucial role of working memory for science learning in elementary school students (Träff et al., 2019; van der Graaf et al., 2016). During the intervention, students’ phonological working memory was challenged by the need to memorize and process task-relevant information, including the written presentation of experimental strategies, the detection of variations between them, and the simultaneous processing of verbal instructions provided by the test administrators (Baddeley, 2012). We did not explicitly manipulate or test the assumption that the phonological working memory component plays an outstanding role due to the text-based nature of the tasks, but our conclusive results are consistent with findings showing the important role of verbal working memory for learning (Friso-van den Bos et al., 2013; van der Graaf et al., 2016).

In line with our hypothesis and previous findings (Cortés Pascual et al., 2019; Rhodes et al., 2014; St Clair-Thompson and Gathercole, 2006), the central executive shows predictive power for students’ performance. Its functions are the allocation of cognitive resources to individual subcomponents and the suppression of distracting stimuli (Baddeley, 2012). The process of focusing attention while simultaneously suppressing irrelevant stimuli is also relevant for the processing of CVS tasks. With regard to content, for example, attention must be directed to the focal variable, and other influencing factors must be kept constant and thus left aside for the moment (Chen and Klahr, 1999).

Finally, the intervention materials and test materials included graphical representations to assist in tracing the experimental set-up. Since students were able to retrieve information from the instructions and task problem in exclusively textual form, we assumed only a limited effect of visuospatial components on learning. Van der Graaf et al. (2016) found in their study that visuospatial working memory shows no link to evidence evaluation. Our results support these findings of this working memory component being no predictor for the learning outcome of CVS. This could be due to the predominantly text-based nature of the tasks, which required more verbal and less visuospatial processing.

Based on findings on contextual grounding and an expertise-reversal effect (Kalyuga, 2007; Son and Goldstone, 2009), we assumed students in the personalized condition to have an advantage in learning over students in the control condition, especially when the latter have a low working memory capacity (Research Question 3). We hypothesized that the students in the personalized condition would benefit from the reduction in the cognitive demands of processing new information by using familiar attributes on the surface level in the instructions and intervention materials. Contrary to our assumption, we did not find evidence to support the moderation effect of the use of context-personalized tasks on the relationship between working memory and students’ performance. Thus, our data do not support theoretical assumptions or empirical evidence of either a learning-enhancing or distracting effect of context personalization on learning the CVS. The results are in line with previous studies that also find null effects of context personalization on students’ performance (Bates and Wiest, 2004; Høgheim and Reber, 2015). Both students in the personalized and the control conditions attended our child-centered intervention sessions with guided instructions that differed only in their contextual features. It is conceivable that personalized tasks, as a further facilitator of the cognitive demands, were not decisive and thus showed no effect of interaction. Another potential explanation for the findings relates to the implementation of Walkington and Hayata’s (2017) design dimensions. Previous studies that yielded null effects typically implemented context personalization on a superficial level, whereas we anticipated our context personalization to be at a medium level and thus having potential for learning-enhancing effects. We manipulated the grain size of the interest-based context in the conditions and surveyed its implementation using the interest questionnaire. We implemented student involvement (ownership) by collecting information on children’s interests and carried out the grouping according to their strongest interests. However, we did not survey students on the extent to which they felt involved in the process of designing the context personalization or the extent to which they perceived it to be authentic in relation to their subject of interest (depth). It is conceivable that the design dimensions need to be better aligned to enable grounding.

### 6.1 Limitations of the present study

The primary limitation of this study lies in the relatively small overall sample size, which resulted in a limited number of clusters and unequal group sizes between the personalized intervention and control conditions. Given the prevalence of absences due to illness in the context of regular school life, a considerable proportion of missing data was observed. This aspect must be considered when interpreting the findings. However, under the assumption that the absences were random and employing robust multiple imputation methods (Rhoads, 2012), potential bias was minimized.

Furthermore, it is important to highlight that conducting the study within a school context, where participants are exposed to a familiar school environment and interact with known peers, enhances the ecological validity of the study (Schmuckler, 2001).

Another limitation arises from the use of a global working memory score in the moderation analysis, which may have obscured component-specific effects. Nevertheless, this decision is theoretically defensible, as working memory operates as an integrated system in which phonological, visuospatial, and executive processes jointly support learning (Baddeley, 2000).

To address the issue of few clusters, we employed cluster-robust standard errors (Huang and Li, 2022) in the statistical analyses; yet, this approach does not fully address potential error accumulation across measurements, which remains a minor limitation due to the small number of measurement points. Despite the use of statistically appropriate procedures, the statistical power of the analyses remains limited.

Moreover, it would be crucial to assess individual interest in the domain of science, as it serves as a predictor of interest in the learning content or the task at hand. To avoid potential biases due to subject-related expectations, we deliberately avoided framing the task as being related to science. Instead, the task was introduced as a riddle. This strategy aimed to ensure that students engaged with the content in a neutral way, uninfluenced by pre-existing dispositions towards science. Finally, it is important to acknowledge that the test administrators interacted with the children during the intervention sessions, raising the possibility of experimenter effects (Rosenthal and Rosnow, 2008). To address this concern, we conducted a comprehensive two-day training program with all test administrators that included role-playing exercises and feedback sessions to enhance their ability to maintain consistency and standardization throughout the study. For future studies, recording the intervention sessions on video could further enhance the ability to verify the consistency of sessions across the small groups.

### 6.2 Implications and future research

Despite the aforementioned limitations, the present study contributes important evidence on effects of context personalization in early science education. Early science education is essential because it establishes the foundation for everyday problem-solving and critical thinking, ultimately preparing children to navigate an increasingly complex world shaped by scientific and technological advancements (National Research Council, 2012). German elementary school curricula at the federal state level contain guidelines for lifeworld-oriented teaching aimed at engaging students and sustaining their interest in science topics, ultimately encouraging their pursuit of science studies and careers (Anders et al., 2018).

We recommend the use of context-personalized tasks for their motivational benefits. By tailoring tasks to resonate with students’ interests and real-world contexts, teachers can potentially enhance students’ declining interest in science (Tröbst et al., 2016). However, the implementation of these tasks without technical support poses significant challenges for teachers. Collaborating with colleagues, leveraging existing educational technologies, and seeking external support can help mitigate resource constraints while maximizing the benefits of personalized learning experiences. Encouraging student involvement in the design process of context-personalized tasks can enhance their sense of ownership and engagement with learning materials while also alleviating stress on teachers. Collaborative efforts among teachers, instructional designers, and researchers can further refine approaches to context personalization, ensuring alignment with educational goals and student needs. Exploring AI-driven platforms or tools that facilitate adaptive learning environments may enhance the feasibility and effectiveness of context personalization in diverse classroom settings (Powell and Courchesne, 2024; Wei et al., 2021; Zhai, 2023). Therefore, in addition to assessing teachers’ resources and attitudes toward interest-based learning (Pannek et al., 2015), future research should investigate AI’s potential role in facilitating contextual personalization.

Finally, given the limited body of research on context personalization in elementary school populations, future studies should explore its effects across various school subjects while systematically addressing all design dimensions, including richness (Walkington and Hayata, 2017).

### 6.3 Conclusion

Elementary school students bring a wide range of interests combined with specific cognitive and motivational potentials to the classroom (Pruisken, 2005). This offers various opportunities for didactic approaches such as context personalization. The present study highlights that context personalization can foster motivational variables, such as situational interest in the learning content. Beyond its short-term effect in a specific learning situation, context personalization has the power to promote the development and maintenance of individual interest in curriculum-relevant topics. Although our data support the central role of working memory components in learning scientific concepts at the elementary school level, we do not find evidence that context personalization is a facilitator for these domain-general cognitive abilities. Although this does not rule out the possibility of interactions with other cognitive variables, we assume that learning-enhancing effects of context personalization are triggered mainly by affective-motivational processes. From this perspective, the use of context personalization in science education can be recommended for students of elementary school age.

## Data Availability

The raw data supporting the conclusions of this article will be made available by the authors, without undue reservation.
